# Measuring critical transitions in financial markets

**DOI:** 10.1038/s41598-017-11854-1

**Published:** 2017-09-14

**Authors:** Jan Jurczyk, Thorsten Rehberg, Alexander Eckrot, Ingo Morgenstern

**Affiliations:** 1 Department of Physics, University of Regensburg, Regensburg, Germany; 2 Institute for Functional Genomics, University of Regensburg, Regensburg, Germany

## Abstract

Tipping points in complex systems are structural transitions from one state to another. In financial markets these critical points are connected to systemic risks, which have led to financial crisis in the past. Due to this, researchers are studying tipping points with different methods. This paper introduces a new method which bridges the gap between real-world portfolio management and statistical facts in financial markets in order to give more insight into the mechanics of financial markets.

## Introduction

Understanding the links between different assets, economic sectors or even between economic zones gives the opportunity to uncover the internal structures of financial markets. These interactions are part of the complex mechanics, which drive prices at stock exchanges up or down^1^. A striking effect in observed price time series are *sudden trend switches* ^1–5^, which sometimes affect only a few assets, while at other times a whole market is impacted and moves in a synchronised fashion. Such turmoils are not limited to stock markets but might have ripple effects on the economy of a country or even of the whole world^6^.

These *tipping points* are an interdisciplinary phenomenon found in fields with complex networks e.g. physics, biology and climate research^7, 8^. Scheffer *et al*.^8^ describe two possibilities of a regime shift. The first results from an exogenous shock while the other one is endogenous. Especially in financial systems connectivity and homogeneity play a major role in crisis research. A small perturbation can have a cascading effect threatening the whole system. When approaching an endogenous tipping point one can observe *critical slowing down*, meaning a perturbation to the system state takes a long time to revert back to the preferred local state.

Hence it is of great interest to develop tools in order to identify current market states.

A common method at the disposal of researchers is the use of the correlation matrix between the log-return time-series of assets traded at stock markets. By using Random Matrix Theory (RMT) on such a correlation matrix it has been shown that markets consist of a dominate market mode and different economic sectors, which exhibit common trends^9–19^. It has been found that the role of the market mode in emerging markets is more important than in developed economic zones where in comparison the clustering of sectors is more dominant^16, 20, 21^.

With the financial crisis of 2008 the focus of researchers shifted to the empirical measurement of systemic risk^22^. In 2010, Billio *et al*.^23^ used Principal Component Analysis (PCA) on the correlation matrix derived from the return series with a sliding time window. They showed that for hedge funds, banks, brokers and insurance companies an increasing entanglement was present during the time of crisis in 2008^23, 24^. This showed the prominent role of these market participants.

Following this train of thought, Zheng *et al*.^22^ used the eigenvectors of the time dependent correlation matrix consisting of ten US sector indices. They found that by tracing the sum of the four largest eigenvalues over time, one can relate the steepest increase to the systemic risk of the US economy represented by the chosen sector indices. Furthermore, they outlined that the magnitude of an increase corresponds to the interconnectedness of the system and therefore provided a precursor indicator.

In this paper, we introduce a new method which links known empirical facts to portfolio management research in order to empirically quantify market states in the S&P 500.

We start with the assumption, that only a few big market participants are responsible for the major changes in share prices^25, 26^ and we assume that these investors are not *noise traders* ^27^ but follow the principle of risk reduction by diversification^28–30^ and belong to the group of fundamental traders^31^. The general idea of a diversified portfolio is that it is more robust against turbulences since not all selected assets are influenced by the news regarding one particular sector^1, 32^. However, determining the correlation between assets faces the problem that time-series in financial markets react on exogenous news and are therefore not stable^33–36^ as commonly assumed^1, 37^. This forces investors to rebalance their portfolio to keep up with the changes within the market. One can imagine the inverse of rebalancing need as the energy barrier, which prevents an investor from moving to the next energy basin and a new preferred state. This paper aims to measure these critical transition points in time.

## Results

Extending the approach by Münnix *et al*.^38^, who investigated the similarity between correlation matrices at different times, we define the similarity between two points in time *t*, *t*′ as the *L*_1_-norm similarity.

1 $$\zeta (t,t^{\prime} )=\frac{2-\parallel {\bf{p}}(t)-{\bf{p}}(t^{\prime} ){\parallel }_{1}}{2}$$

between two portfolios P(t), P(t′). The entries of the similarity matrix *ζ* effectively measure the transaction costs an investor has to pay in order to rebalance the portfolio from one point in time to another. We use the daily closing prices of the S&P 500 components from 2000 until the end of 2016 as input data for the creation of mean-variance portfolios with a time window *ω* of 3 years.

Choosing the parameter *ω* to be 3 years ensures that the ratio between the number of data-points and the number of assets *Q* is greater than 1.5 and therefore the correlation matrix is always well behaved.

**Figure 1:**
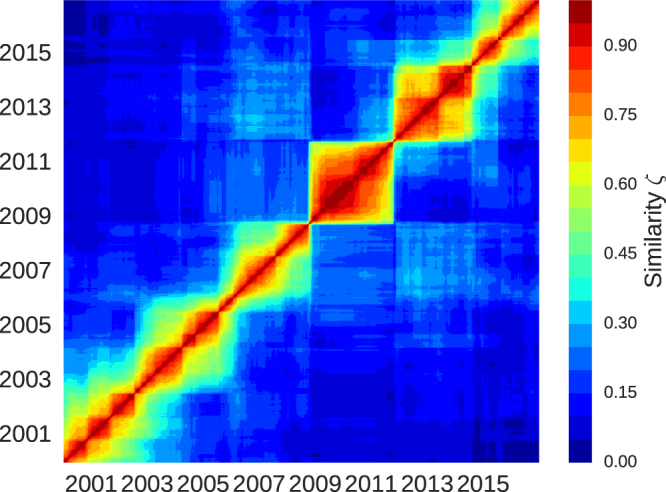
Similarity matrix *ζ* for the minimal variance investor approximation. There is a clear separation in October 2008 and again in October 2011. The subclusters are also a sign of minor changes within the minimum-variance portfolios.

Figure (1) shows the similarity matrix *ζ* for the minimal variance portfolios.

In this matrix, one can identify two clusters of high similarity. The first one spans from October 2008 to September 2011 with an average similarity *ζ* = 0.58. The next visible cluster ranges from September 2011 until July 2014 and has an average similarity of *ζ* = 0.48. Before October 2008, the high similarity clusters are overlapping each other and do not show such a sharp structural change as in October 2008, September 2011 and July 2014. One notices smaller transitions, for example in September 2001, but these occur within a moderate similarity level.

In order to get more detailed information on the market phase duration depicted by similarity clusters, we use eigenvalue decomposition on the similarity matrix *ζ*.

The resulting normed eigenvalues ${\lambda }_{0}\mathrm{ > ... > }\,{\lambda }_{k}\mathrm{ > ... > }\,{\lambda }_{{k}_{max}}$ correspond to the importance of the eigenvector **u**_*k*_ in decreasing order.

These unit eigenvectors are orthogonal to each other and describe the data with a new set of basis vectors. In our case, the eigenvectors have the following interpretation: Each component of an eigenvector corresponds to a point in time where its absolute value signals whether at time *t* there is a significant contribution for describing a certain level of similarity in the matrix *ζ*. Since the eigenvectors correspond to a time-series, successive high values in the eigenvector imply a similar level of similarity. Therefore by following the temporal evolvement of *u*_*kt*_, one follows a direction of similarity level. By taking the fourth power of every entry ${u}_{kt}^{4}$, unimportant entries become negligible small while the rest are amplified, which is usually done when calculating the inverse participation ratio^39, 40^. This allows us to capture the participation of the eigenvector to a market state at that point in time.

**Figure 2:**
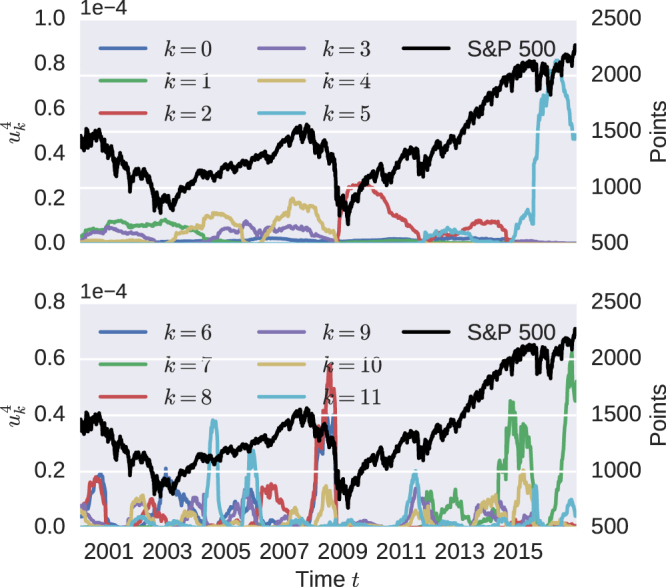
The fourth power of the first twelve eigenvectors ${{\bf{u}}}_{k}^{4}$ for the minimum-variance similarity matrix (*γ* = 0) in comparison to the S&P 500 Index (black line). There are several transition present from 2000–2017. For example, the high similarity between portfolios are mainly described by eigenvectors k = 6, 8, 10.

In Fig. (2) the first twelve eigenvectors to the fourth power are shown, which cover approximately 82% of the information.

The eigenvectors *k* = 1, 3 and 4 are needed to explain most of the similarities between 2000 and 2008 and represent 28% of the overall information. The eigenvectors *u*_2_ and *u*_5_ are representing 17% of the similarity matrix. One notices the separation in October 2008. After this point in time a new subspace is needed to describe the properties of the similarity matrix *ζ*. This is a sign of structural change in the investor’s minimal-variance portfolio. From October 2008 on, an investor would have had to completely change his portfolio in order to reposition himself in the new market environment. The next six eigenvectors show shorter clusters. In the subspace spanned by the directions of *k* = 6, 8, 10 one can find a cluster right before the financial crisis of 2008. This phase ranges from November 2007 until October 2008. All other eigenvectors have a significant participation over the whole period of time from 2000 until 2017 and are showing shorter phases with equal similarities, except the eigenvector *u*_7_.

Since this analysis relies on visually inspecting the temporal development of the eigenvectors, we automatized this procedure by using an algorithm (see methods), which maps, from a chosen set of eigenvectors, the significant ones at a time *t* to a state.

**Figure 3:**
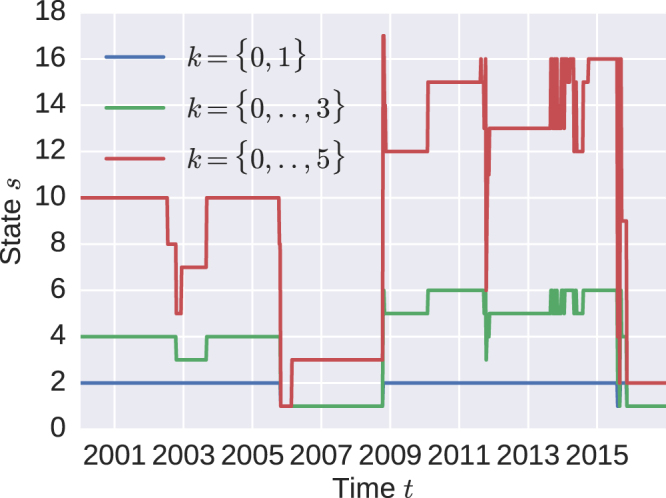
States for the minimal variance investor for different sets of eigenvectors. The eigenvectors *k* = 0, 1 cover 39%, *k* = 0, ..., 3 cover 57% and *k* = 0, .., 5 make up 71% of the similarity matrix information.

In Fig. (3) the result of this algorithm for *k* = {0, 1}, *k* = {0, 1, 2, 3} and *k* = {0, 1, 2, 3, 4, 5} is shown. A lower state indicates that eigenvectors with smaller eigenvalues are used to construct this state.

If only the first two eigenvectors are used, the S&P 500 only uses two out of the 2^2^ possible states, which means at least one eigenvector is significant over the whole time. State *s* = 2 lasts from 2000 until the end of October 2005, after that *s* = 1 is occupied until October 2008. Between July 2015 and November 2015 the market is switching from *s* = 1 to *s* = 2 five times and settles in state *s* = 1 after that.

Increasing the number of eigenvectors also increases the number of possible states but the number of actual occupied states is much lower than the number of possible states (2^|*k*|^). The biggest changes in these three cases occur in October 2005, October 2008, October 2011 and July 2015.

In order to verify whether there is an impact on the S&P 500 by a state change, we performed a Granger causality test^41^ between the changes in overall trading volume within a week and the absolute weekly state changes.

The results in Table 1 show that the week after a state change, with more than two eigenvectors, is linked to the volume changes in the S&P 500. For lags longer than a week, there is no Granger causality on a 0.05 significance level performed with a F-test.

**Table 1:** Results of the Granger Causality p-values for four different sets of eigenvectors with up to five weeks of lag.

k=	{0, 1}	{0, .., 3}	{0, .., 5}	{0, .., 11}
1*w*	0.12	0.01*	0.03*	0.03*
2*w*	0.06	0.07	0.09	0.07
3*w*	0.21	0.21	0.26	0.2
4*w*	0.36	0.14	0.15	0.13
5*w*	0.22	0.12	0.13	0.11

The p-values denoted with a * show a p-value below 0.05.

## Discussion

The approximation of a risk aware investor by the mean-variance model aims to close the gap between stylized facts (volatility clustering, fat tails) and the connection between systemic risks and correlation matrices.

The S&P 500 analysis shows similar transition points to the pure PCA analysis of a correlation matrix^22, 24, 42^. From 2000 until 2017, there is clear jump in 2008 in all investor approximations *γ*∈[0, 1] supporting research by Preis *et al*.^1^, who found the *diversity breakdown* in times of market stress.

That implies that one can measure the transition point *t*_*c*_ of a financial system by applying eigenvalue decomposition on the similarity matrix *ζ* and determine whether *t*′ belongs to the same similarity subspace as *t* < *t*′. If at *t*′ another set of eigenvectors is needed, then *t*′ is a transition point *t*_*c*_. By increasing the number of eigenvectors and therefore gaining descriptive information of the similarity matrix, more transition points can be identified and more subtle distinctions between states can be found. These points in time highlight the investment shifts in the market, which can be small or larger structural changes as it was the case in 2008 and 2011. These critical transitions are linked to volume changes in the S&P 500.

In summary, our method to find critical transition points within a financial market is based on the similarity between mean-variance portfolios at different times. The resulting matrix is analysed by eigenvalue decomposition, which uncovers the temporal development of different similarity levels. As a last step, the subspaces formed by the eigenvectors are mapped to unique states along the time axis. This method allows to find two new aspects of financial markets: Market phases must be classifiable by an investor approximation, times of systemic risk fall into transition phases. These results are complementary to the known stylized facts and should be incorporated when constructing new general financial market models.

## Methods

In order to approximate an investors risk aversion, we use the mean-variance model by Markowitz^43, 44^ in its classical form. This is an optimisation problem, where one has to minimise the variance and maximise the return of a portfolio **p**. Moreover, by restricting the portfolio components *p*_*i*_ to be positive for all available assets *N*, we only allow long positions. The resulting selection problem is solved by minimising the cost-function

2 $$-\gamma \sum _{i=1}^{N}{\mu }_{i}{p}_{i}+\mathrm{(1}-\gamma )\sum _{i,j}^{N}{p}_{i}{C}_{ij}{p}_{j}$$

where *μ*_*i*_ is the expected return of asset *i* and **C** the pearson covariance matrix. The parameter *γ* is used to balance the trade-off between risk and return. A value of *γ* = 0 would result in only minimising the variance, while a value of *γ* = 1 would cause portfolios to be only optimized for maximum return.

We implemented a coordinate descent algorithm as used by Friedman *et al*.^45, 46^ for fitting generalised linear models in order to generate efficient portfolios for a specific *γ*. As proposed by Altenbuchinger *et al*.^47^, one can incorporate the equality constraint ${\sum }_{i}{p}_{i}=1$ in the coordinate descent algorithm by substituting ${p}_{k}=1-{\sum }_{ii\ne k}{p}_{i}$. By calculating the partial derivative and solving for *p*_*k*_ one obtains an extreme value for *p*_*k*_ and *p*_*s*_. With the help of the second derivative and curve sketching, one can fulfill the constraint *p*_*i*_ ≥ 0 and a coordinate descent step can be constructed. Iterating this update step in combination with active set cycling^46, 48, 49^, allows the generation of thousands of portfolios in a reasonable time frame.

The algorithm for mapping a given set of eigenvectors to a state works in 4 steps:

1. Calculate the participation ${\rm{U}}={u}_{kt}^{4}$ and normalize each column to the max-Norm.
2. Map U to a state matrix S = U > *τ*, where *τ* is a given threshold. *τ* is set to 0.01 in this paper. Each row of S now represents a state.
3. These states are now mapped to a state number S → *s* between 1 and the number of unique states found in S, where state 1 is the state combined from the eigenvectors with the lowest eigenvalues.
4. The state time-series **s** is returned.

The data we used to create the mean-variance portfolios was downloaded from the WIKI Quandl database^50^.The financial time-series data for the S&P 500 was downloaded with the QUANDL50 data interface from the WIKI database.

## References

[1] Preis T, Kenett DY, Stanley HE, Helbing D, Ben-Jacob E (2012). Quantifying the behavior of stock correlations under market stress. Scientific reports.

[2] Petersen, A. M., Wang, F., Havlin, S. & Stanley, H. E. Market dynamics immediately before and after financial shocks: Quantifying the Omori, productivity, and Bath laws. *Physical Review E - Statistical, Nonlinear, and Soft Matter Physics* **82** (2010). 10.1103/PhysRevE.82.036114 21230146

[3] Preis T, Schneider JJ, Stanley HE (2011). Switching processes in financial markets. Proceedings of the National Academy of Sciences of the United States of America.

[4] Preis T, Stanley HE (2010). Switching phenomena in a system with no switches. Journal of Statistical Physics.

[5] Preis T (2011). Econophysics—complex correlations and trend switchings in financial time series. The European Physical Journal-Special Topics.

[6] Balogh, E., Simonsen, I., Nagy, B. Z. & Néda, Z. Persistent collective trend in stock markets. *Physical Review E - Statistical, Nonlinear, and Soft Matter Physics* **82** (2010). 10.1103/PhysRevE.82.066113 21230711

[7] Scheffer M (2009). Early-warning signals for critical transitions. Nature.

[8] Scheffer M (2012). Anticipating critical transitions. Science (New York, N.Y.).

[9] Bouchaud, J. P. & Potters, M. Financial applications of random matrix theory: a short review. *arXiv* 0910.1205 (2009).

[10] Potters M, Bouchaud JP, Laloux L (2005). Financial applications of random matrix theory: Old laces and new pieces. In. Acta Physica Polonica B.

[11] Rosenow B, Plerou V, Gopikrishnan P, Amaral LAN, Stanley HE (2000). Application of Random Matrix Theory To Study Cross-Correlations of Stock Prices. International Journal of Theoretical and Applied Finance.

[12] Utsugi, A., Ino, K. & Oshikawa, M. Random matrix theory analysis of cross correlations in financial markets. *Physical Review E - Statistical, Nonlinear, and Soft Matter Physics* **70** (2004). 10.1103/PhysRevE.70.026110 15447548

[13] Kenett, D. Y., Shapira, Y. & Ben-Jacob, E. RMT assessments of the market latent information embedded in the stocks’ raw, normalized, and partial correlations. *Journal of Probability and Statistics* (2009).

[14] Plerou V, Gopikrishnan P, Rosenow B, Nunes Amaral L, Stanley H (1999). Universal and Nonuniversal Properties of Cross Correlations in Financial Time Series. Physical Review Letters.

[15] Plerou, V. *et al*. Random matrix approach to cross correlations in financial data. *Physical Review E - Statistical, Nonlinear, and Soft Matter Physics* **65** (2002). 10.1103/PhysRevE.65.066126 12188802

[16] Shen J, Zheng B (2009). Cross-correlation in financial dynamics. EPL (Europhysics Letters).

[17] Laloux, L. & Bouchaud, J.-P. Random Matrix Theory and Financial Correlations. *International Journal of Theoretical and Applied Finance* **3** (2000).

[18] Laloux L, Cizeau P, Bouchaud J-P, Potters M (1999). Noise Dressing of Financial Correlation Matrices. Physical Review Letters.

[19] Qiu T, Zheng B, Chen G (2010). Financial networks with static and dynamic thresholds. New Journal of Physics.

[20] Pan, R. K. & Sinha, S. Collective behavior of stock price movements in an emerging market. *Physical Review E - Statistical, Nonlinear, and Soft Matter Physics* **76** (2007). 10.1103/PhysRevE.76.046116 17995069

[21] Jiang XF, Zheng B (2012). Anti-correlation and subsector structure in financial systems. EPL (Europhysics Letters).

[22] Zheng Z, Podobnik B, Feng L, Li B (2012). Changes in cross-correlations as an indicator for systemic risk. Scientific reports.

[23] Billio, M., Getmansky, M. & Lo, A. Measuring systemic risk in the finance and insurance sectors. *NBER working paper* (2010).

[24] Billio M, Getmansky M, Lo AW, Pelizzon L (2012). Econometric measures of connectedness and systemic risk in the finance and insurance sectors. Journal of Financial Economics.

[25] Coyne, K. P. & Witter, J. W. What makes your stock price go up and down. *McKinsey&Company* Number 2 (2002).

[26] Keim DB, Madhavan A (1995). Anatomy of the trading process empirical evidence on the behavior of institutional traders. Journal of Financial Economics.

[27] Wang F (2001). Overconfidence, Investor Sentiment, and Evolution. Journal of Financial Intermediation.

[28] Meucci A (2010). Managing Diversification. Risk pp 7479 May 2009.

[29] Fragkiskos, A. What is Portfolio Diversification? *Alternative Investment Analyst Review* 8–18 (2014).

[30] Cesarone, F., Moretti, J. & Tardella, F. Does Greater Diversification Really Improve Performance in Portfolio Selection? *Available at SSRN 2473630* 1–15 (2014).

[31] Lux T, Marchesi M (1999). Scaling and criticality in a stochastic multi-agent model of a financial market. Nature.

[32] Eckrot A, Jurczyk J, Morgenstern I (2016). Ising model of financial markets with many assets. Physica A: Statistical Mechanics and its Applications.

[33] Brock W, Hommes CH, Wagener F (2009). More hedging instruments may destablize markets. Journal of Economic Dynamics & Control.

[34] Kenett DY, Preis T, Gur-Gershgoren G, Ben-Jacob E (2012). Dependency Network and Node Influence: Application To the Study of Financial Markets. International Journal of Bifurcation and Chaos.

[35] Kenett, D. Y., Raddant, M., Lux, T. & Ben-Jacob, E. Evolvement of uniformity and volatility in the stressed global financial village. *PLoS ONE* **7** (2012). 10.1371/journal.pone.0031144 PMC3275621 22347444

[36] Kenett, D. Y. *et al*. Index cohesive force analysis reveals that the US market became prone to systemic collapses since 2002. *PLoS ONE* **6** (2011). 10.1371/journal.pone.0019378 PMC3083438 21556323

[37] Campbell RAJ, Forbes CS, Koedijk KG, Kofman P (2008). Increasing correlations or just fat tails?. Journal of Empirical Finance.

[38] Münnix MC (2012). Identifying states of a financial market. Scientific Reports.

[39] Rosenow B, Plerou V, Gopikrishnan P, Stanley HE (2002). Portfolio Optimization and the Random Magnet Problem. Europhysics Letters.

[40] Conlon T, Ruskin HJ, Crane M (2007). Random matrix theory and fund of funds portfolio optimisation. Physica A: Statistical Mechanics and its Applications.

[41] Granger C (1980). Testing for causality. Journal of Economic Dynamics and Control.

[42] Kritzman M, Li Y, Page S, Rigobon R (2011). Principal Components as a Measure of Systemic Risk. The Journal of Portfolio Management.

[43] Markowitz H (1952). Portfolio Selection*. The J. Finance.

[44] Markowitz, H. M. Portfolio Selection: Efficient Diversification of Investments (1959).

[45] Friedman J, Hastie T, Höfling H, Tibshirani R (2007). Pathwise coordinate optimization. The Annals of Applied Statistics.

[46] Friedman JH, Hastie T, Tibshirani R (2010). Regularization paths for generalized linear models via coordinate descent. Journal of Statistical Software.

[47] Altenbuchinger M (2017). Reference point insensitive molecular data analysis. Bioinformatics.

[48] Krishnapuram B, Carin L, Figueiredo MA, Hartemink AJ (2005). Sparse multinomial logistic regression: Fast algorithms and generalization bounds. Pattern Analysis and Machine Intelligence, IEEE Transactions on.

[49] Meier L, Van De Geer S, Bühlmann P (2008). The group lasso for logistic regression. Journal of the Royal Statistical Society: Series B (Statistical Methodology).

[50] Quandl. Quandl Financial and Economic Data.

